# Burkitt-like lymphoma with 11q aberration in a patient with AIDS and a patient without AIDS: Two cases reports and literature review

**DOI:** 10.1515/med-2021-0246

**Published:** 2021-03-16

**Authors:** Jing Wang, Li Ma, Jianghong Guo, Yanfeng Xi, Enwei Xu

**Affiliations:** Department of Pathology, Shanxi Tumor Hospital, No. 3, Kaixuan Street, Xinghualing District, Taiyuan City, Shanxi Province, China; Department of Hematology, Shanxi Tumor Hospital, No. 3, Kaixuan Street, Xinghualing District, Taiyuan City, Shanxi Province, China

**Keywords:** Burkitt-like lymphoma with 11q aberration, Burkitt lymphoma, MYC rearrangement, pathology

## Abstract

**Objective:**

The aim of this study is to evaluate the clinicopathological features and the treatment of the Burkitt-like lymphoma with 11q aberration.

**Methods:**

We reported two patients with Burkitt-like lymphoma with 11q aberration: a 56-year-old man with AIDS (case 1) and a 37-year-old woman (case 2) without AIDS. The biopsy of cervical lymph nodes showed Burkitt-like morphologic and immunophenotypic features. But both of them lack MYC rearrangement and carry an 11q-arm aberration with proximal gains and/or telomeric losses. The diagnosis was confirmed by pathological morphology, immunohistochemistry, and fluorescence *in situ* hybridization.

**Result:**

After a cycle of R-CTOEP (rituximab, cyclophosphamide, pirarubicin, vincristine, and prednisone) chemotherapy, case 1 refused to chemotherapy and radiotherapy and was followed up for 34 months without recurrence and new focus. Case 2 received R-CHOP (rituximab, cyclophosphamide, doxorubicin, vincristine, and prednisone) for two cycles and achieved PR (partial response). Then, the patient in case 2 received EPOCH (etoposide, prednisone, vincristine, cyclophosphamide, and doxorubicin) for three cycles, and the right cervical mass disappeared. She achieved complete response and was followed up for 16 months without recurrence and new focus.

**Conclusion:**

Burkitt-like lymphoma with 11q abnormalities resembles Burkitt lymphoma morphologically but lacks MYC rearrangement and may have a better prognosis.

## 1 Introduction

The revised World Health Organization (WHO) Classification of Haematopoietic and Lymphatic Tissues published in 2017 had significant changes from the previous edition. High-grade B-cell lymphomas (HGBCLs) replaced B-cell lymphoma, unclassifiable, with features intermediate between diffuse large B-cell lymphoma and Burkitt lymphoma. “Burkitt-like lymphoma with 11q aberration” (abbreviated “mnBLL-11q”) was proposed as a provisional subtype. This entity is described as cases that resemble Burkitt lymphoma morphologically and immunophenotypically but lack the characteristic MYC rearrangements and instead show a chromosome 11q alteration characterized by proximal gains and telomeric losses [1]. However, the number of cases harboring this aberration is very limited. Here, we report two cases of mnBLL-11q confirmed by immunohistochemistry and the fluorescence *in situ* hybridization (FISH) test and also review relevant literature to investigate the nature and clinicopathological features and to improve the diagnostic alertness of this rare lymphoma subtype.

## 2 Case description

### 2.1 Case 1

A 56-year-old man was admitted to the Department of Hematology at Shanxi Tumor Hospital (Taiyuan, Shanxi Province, China) for further treatment on January 8, 2018, after a left neck tumor resection at another institution. He found a 1 cm mass in the left neck in August 2017. The tumor subsided after anti-infective treatment, but similar tumors were found again in September. Since any anti-infective treatment was ineffective, he underwent a left neck tumor resection at the local hospital in mid-October. The pathological diagnosis was invasive B-cell lymphoma.

When he came to our hospital for further treatment, the general condition of him was good except a long healed scar of 4 cm on the left neck. Examination by a specialist showed multiple enlarged lymph nodes in the left neck, and the largest was 2 cm × 3 cm. There was no tenderness, no swelling of superficial lymph nodes, and no hyperemia of the pharynx except slight swelling of the left face. He has clear respiratory sounds of both lungs and a uniform heart rate of 94 beats per minute. His abdomen was soft and has no mass, no tenderness, and rebound pain. The liver and spleen were not subcostal touched. There was no edema in the lower limbs.

He has a history of hepatitis for 10 years and had HIV infection 1 year ago. He was on HAART (compound sulfamethoxazole, ganciclovir, and fluconazole), and his AIDS was well controlled. He denied the history of blood transfusion and the history of food and drug allergy. The laboratory tests presented 75.2% total T cells, 12.9% helper T cells, 60.2% cytotoxic T cells, and 5.9% of total B-cells.

**Figure 1:**
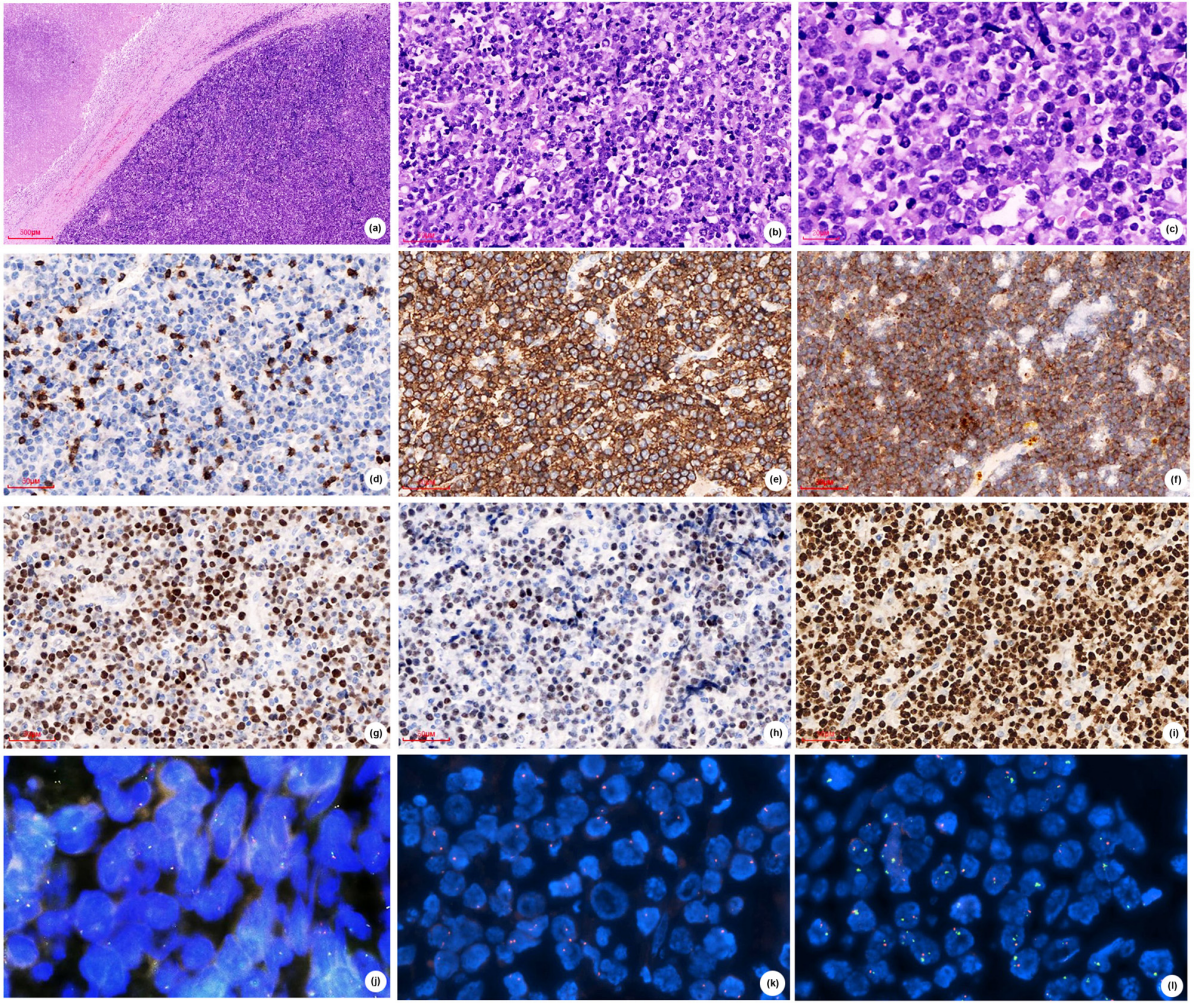
Case 1 (A), (B) H&E stained sections show diffuse medium-big sized cells with (C) thick nuclear membrane, rough chromatin, frequent apoptotic bodies and mitoses, forming a "star-sky phenomenon". Tumor cells are negative for CD3 (D), positive for CD20 (E), CD10 (F) and Bcl-6 (G), and are negative for c-MYC (H), has high proliferation rate by Ki‐67 (I), (A,×50; B,D-J,×200; C,×600). FISH showed negative for MYC rearrangements (J) and positive for centromeres lost (K) and 11q aberration (L).

To determine the type of his invasive B-cell lymphoma, he borrowed formalin-fixed paraffin-embedded (FFPE) tissues from the hospital where the operation was performed. The immunohistochemical and molecular examinations were performed in the Department of Pathology of Shanxi Tumor Hospital. Microscopic morphology showed that the normal structure of the lymph node disappeared and was replaced by diffuse medium- to big-sized cells with a thick nuclear membrane, rough chromatin, frequent apoptotic bodies, and mitoses (Figure 1a and b). The phagocytes scattered among the cells engulfed nuclear fragments, forming a “star-sky phenomenon” (Figure 1c). Immunohistochemistry was positive for CD20 (Figure 1e), CD10 (Figure 1f), and Bcl-6 (Figure 1g) and negative for BCL-2. The Ki-67 proliferation index was more than 90% (Figure 1i). CD3 (Figure 1d) and c-MYC (Figure 1h) were negative. Although both morphology and immunohistochemistry showed a Burkitt-like pattern, *in situ* hybridization (FISH) was negative for MYC (Figure 1j) rearrangements, as well as BCL-2 and BCL-6, and Epstein–Barr virus (EBER) testing was negative. We suspected it as mnBLL-11q defined by the revised 2016 WHO of lymphoid neoplasms, so we sent the FFPE tissues to Professor John K. C. Chan, pathologist of Department of Pathology, Queen Elizabeth Hospital, Hong Kong. In this case, centrosomes loss (Figure 1k) and 11q aberration (Figure 1l) were detected by FISH, and Professor John K. C. Chan confirmed mnbl-11q.

After a cycle of R-CTOEP (rituximab, cyclophosphamide, pirarubicin, vincristine, and prednisone) chemotherapy, he refused further chemotherapy. He was followed up for 34 months in good condition without recurrence and new focus.

### 2.2 Case 2

A 34-year-old young woman was admitted to the Department of Hematology at Shanxi Tumor Hospital (Taiyuan, Shanxi Province, China) for right neck mass on July 27, 2019. She found a 3 cm × 3 cm neck mass after a toothache, a month ago. Anti-inflammatory treatment was carried out, but the effect was poor, and the neck mass grew rapidly. The size of the tumor was about 10 cm × 10 cm at the time of admission. She presented with neck pain, poor breathing, swallowing, numbness on the right lingual side, hoarseness, cough, expectoration, and limited mouth opening.

She was physically healthy, and there was no history of hypertension, diabetes, and heart disease. Also, she has no history of hepatitis, tuberculosis, and other infectious diseases (HBV [−], HCV [−], HIV [−], EBV [−]). She denied the history of blood transfusion and the history of food and drug allergy. Her family history included lung cancer in her mother who died at an unknown age.

Her vitals were within a normal range. The examination by specialist was normal except 12 cm × 10 cm mass of the right neck, which was hard and poorly moved. The surface of the skin was red and swollen. She felt pain when the doctor pressed the nodule on her right neck. There was no palpation and enlargement of the thyroid lobes. The superficial lymph nodes of the whole body were not touched and enlarged.

The results of nasopharyngoscope and electronic fiberoptic laryngoscope showed that there were no new organisms and abnormal secretions in the nasopharynx. She has two swellings of the right tonsil, swelling of the soft palate, and hyperplasia of lymphoid follicles at the root of the tongue. Ultrasonography showed a large solid tumor in the right neck, which is suggestive of a malignant tumor. The plain scan CT of the neck demonstrated a large soft tissue mass in the left cervical space, wrapped around the right internal jugular vein.

**Figure 2:**
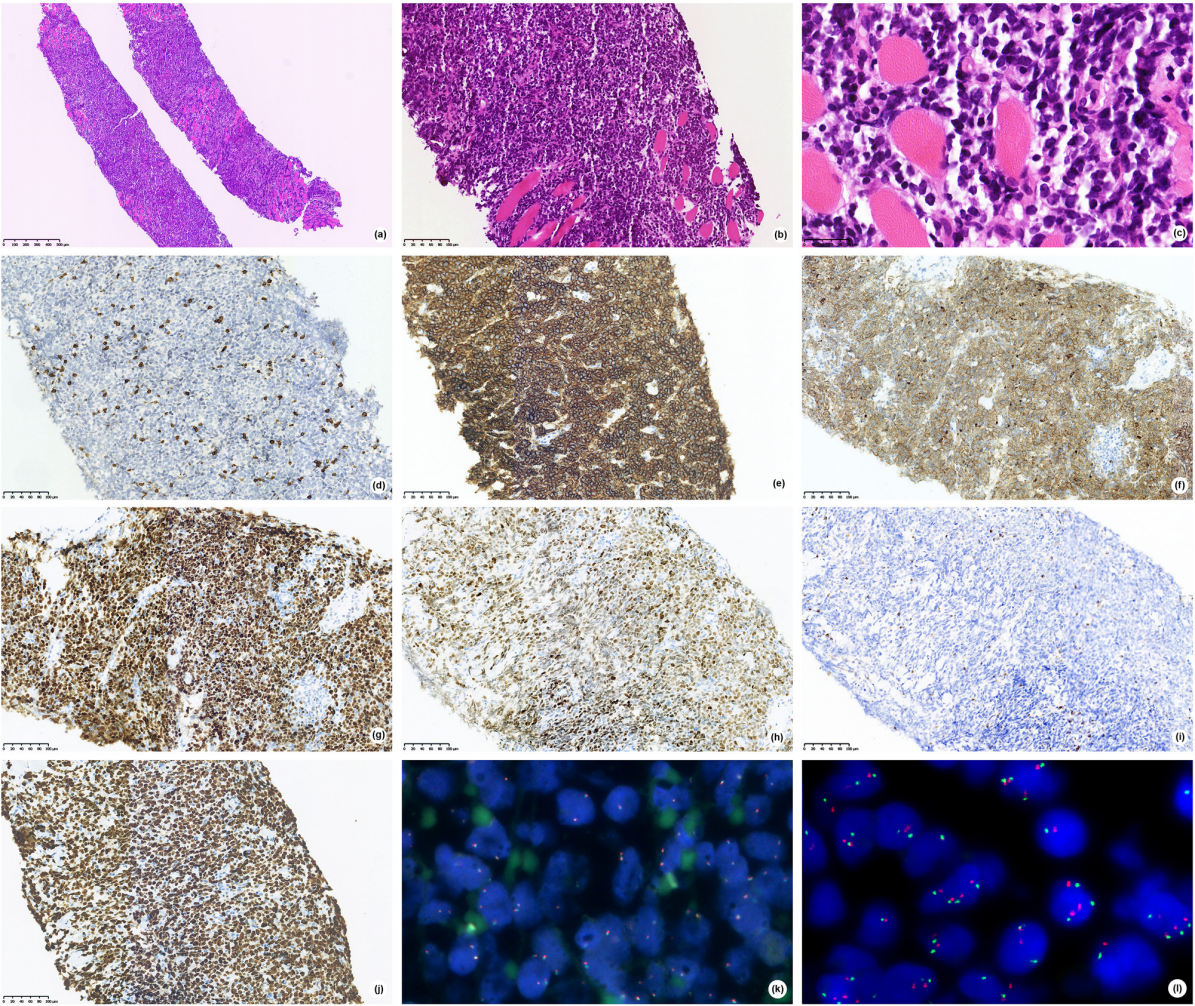
Case 2 (A), (B) H&E stained sections show diffuse medium-sized lymphoid cells with (C) irregular nuclei, fineness chromatin, Minimal to moderate cytoplasm. Tumor cells are negative for CD3 (D), positive for CD20 (E), CD10 (F), Bcl-6 (G) and Mum-1 (H), and are negative for BCL2 (I), has high proliferation rate by Ki‐67 (J), (A,×50; B,D–J,×200; C,×400). FISH showed negative for MYC rearrangements (K)and positive for 11q aberration (L).

With informed consent from the patient, the surgeon proceeded with a puncture biopsy of the tumor in the right neck, which revealed diffuse medium-sized lymphoid cells (irregular nuclei, fineness chromatin, minimal to moderate cytoplasm; Figure 2a–c) and diffuse infiltrate within the striated muscle. Immunophenotypically, the neoplastic cells show diffuse expression for CD20 (Figure 2e), CD10 (Figure 2f), BCL-6 (Figure 2g), and along with very high proliferation rate by Ki-67 (>80%, Figure 2j). Mum-1 (Figure 2h) was positive. CD3 (Figure 2d) and BCL-2 (Figure 2i) were negative. EBER was negative. FISH was negative for BCL-2, BCL-6, and MYC (Figure 2k) rearrangements. 11q aberration was analyzed by FISH using 11q23.3 and 11q24.3 probes. One hundred cells were counted, and the results were 3R3G 10%, 4R3G 4%, 4R4G 17%, 5R5G 3%, and 4R5G 2% (Figure 2l). These findings demonstrated an aggressive mnBLL-11q.

With informed consent from the patient, the surgeon proceeded with a bone marrow puncture and bone marrow biopsy. Biopsy of bone marrow showed normal bone marrow. Flow cytometry (FCM) showed no obvious phenotype abnormality. Cytogenetic analysis showed a normal karyotype. The mutational status was detected by next-generation sequencing (NGS) and showed no gene mutation of 59 genes.

The woman was classified as II-A and aaIPI score 0 (age, ECOG 0 score, LDH, clinical stage, and extra-nodal lesion). She was treated with R-CHOP (rituximab, cyclophosphamide, doxorubicin, vincristine, and prednisone) chemotherapy for two cycles. After her initial chemotherapy, a repeat CT scan showed a significant reduction in the tumor size to 3 cm × 4 cm. Since CT assessment of the condition just showed part relief (PR), she received EPOCH (prednisone, etoposide, doxorubicin, vincristine, and cyclophosphamide) for three cycles, and the right cervical mass disappeared.


**Informed consent:** Informed consent has been obtained from all individuals included in this study.

## 3 Discussion

### 3.1 History

Burkitt lymphoma is defined by WHO as a highly invasive lymphoid tissue tumor characterized by MYC gene rearrangement, diffuse medium-sized lymphoid cells, forming a typical “starry sky” pattern. B-cell markers (such as CD20, CD79a, and PAX5) were co-expressed with germinal center markers (such as CD10 and BCL-6), with a high cell proliferation rate (usually close to 100%) [1]. Recent DNA copy number alteration (CNA) and NGS studies have provided a comprehensive catalog of genomic aberrations in BL [2]. As early as 2011, Pienkowska-Grela et al. [3] have reported four cases of “Burkitt lymphoma” without c-MYC rearrangement and found 11q23q13 duplication, and they were diagnosed as gray area lymphoma with characteristics intermediate between diffuse large B-cell lymphoma and Burkitt lymphoma, and they believed that the identification of such abnormalities would be helpful for diagnosis. This abnormality of chromosome 11 was further confirmed and fully studied in the Salaverria et al. [4] series. By analyzing 17 cases of MYC-negative HGBCLs, Salaverria et al. found that all of them shared a peculiar pattern of chromosome 11q aberration characterized by interstitial gains including 11q23.2-q23.3 and telomeric losses of 11q24.1-qter, and this aberration recurrently associated with morphologic and clinical features of BL. They believed that there is a molecularly distinct subset of B-cell lymphomas, similar to BL, which is characterized by deregulation of genes in 11q. Then, in 2015, Ferreiro et al. [5] reported seven cases of Burkitt-like lymphoma after transplantation and three cases of them with MYC negative and 11q abnormality, and hence, they suggested that such tumors may be related to transplantation and immune deficiency. According to the aforementioned literature, this entity was designated as “Burkitt-like lymphoma with 11q aberration” in the 2017 revision of the WHO classification of lymphoid neoplasms. This entity comprises B lymphocytes, similar to Burkitt lymphoma morphologically, immunophenotypically, and by gene and microRNA expression profiles, but lacking MYC rearrangement. Instead, they carry a complex karyotype including proximal gains and distal losses of the long arm of chromosome 11 [6].

### 3.2 Clinicopathological features

Later, several articles described additional cases around the world [7,8,9,10]. Strobel et al. [9] reported a case of nmBLL-11q in a pediatric patient with familial adenomatous polyposis in 2019. They assumed that the perturbation of the MYC network due to FAP and the 11q aberration may have been co-drivers in the pathogenesis of nmBLL-11q. A summary of the previously reported cases found that they have similar clinicopathological features. Although it can occur in older people [7,10], nmBLL-11q is more common in children and young adults, more in men than women, and more commonly localized at the head and the neck. The histopathology and immunological morphology are very similar to the Burkitt lymphoma as mentioned earlier and lack MYC rearrangement (FISH), instead of 11q aberration (FISH). In the two cases we reported, both appears as a neck nodule; case 1 was a middle-aged patient and has relatively large cells with more apoptotic cells, showing a typical “starry sky” pattern. Case 2 is a young patient and has relatively small cells with irregular nucleus and few apoptotic bodies, and the “starry sky” pattern was not obvious. The masses were all in the neck and had occurred after a history of infection. Some studies suggested that such tumors may be related to transplantation and immune deficiency, as in the posttransplant setting [5]. Case 1 of our study, as well as 1 in 11 cases in the study by Gonzalez-Farre et al. [11], was infected by HIV. But case 2 did not show any immunodeficiency. Both cases we reported tested negative for EBER FISH, which is consistent with previous reports [11].

### 3.3 Molecular characteristics

However, as a provisional subtype, it is debatable whether these lymphomas are a unique type or specific variants of other recognized entities. The 11q-gain/loss was found unspecific for mnBLL-11q because Beata et al. [12] found that the 11q-gain/loss not only occurs in nmBLL-11q but also occurs recurrently in MYC-positive BL and MYC-positive HGBCL, as well as the findings of Havelange et al. [13]. Since a particular imbalance pattern on chromosome 11 has been identified, the picture of mutations has not been described. Recent DNA CNA and NGS studies have provided a set of genomic aberrations in mnBLL-11q [12,14]. The genes *ATM*, *CBL*, *CCND1*, *KMT2A*, and *USP2* are in the minimal gain region, and *FLI1*, *ETS1*, and *ZNF202* genes in the minimal loss region all shown to be involved in tumorigenesis of mnBLL-11q [9]. The coincidence of 11q replication and deletion (resulting from the aforementioned driver mutations) suggests the possibility of simultaneous upregulation of oncogenes and downregulation of tumor suppressor genes. The specific molecular characteristics of mnBLL-11q increase uncertainty about their exact classification as a particular variant of BL, diffuse large B-cell lymphoma, or a distinct form of HGBCL [12,13,14,15]. Wagener et al. [14] investigated 15 MYC-negative mnBLL-11q cases, and they found that the genome map of mnBLL-11q is different from BL in both chromosome and mutation levels since they did not detect recurrent mutations in genes of the ID3-TCF3 axis or SWI/SNF complex, which are frequently altered in Burkitt lymphoma [15]. They also found that *GNA13* gene plays a role in the pathogenesis of mnBLL-11q and identified *NFRKB* gene as a candidate gene in the deleted region in 11q24.3. These findings are in line with observations of Gonzalez-Farre et al. [11]. They believed that mnBLL-11q is a germinal center–derived lymphoma that is closer to high-grade B-cell lymphoma or diffuse large B-cell lymphoma rather than Burkitt lymphoma, and they suggested that the term “aggressive B-cell lymphoma with 11q distortion” is more consistent with the pathological characteristics of this kind of lymphoma than mnBLL-11q.

### 3.4 Diagnosis

Molecular characteristics of this uncommon lymphoma subtype need to be identified and diagnosed because of its special clinical significance, so this diagnosis should be considered in patients with MYC-negative high-grade B-cell non-Hodgkin’s lymphoma, especially in younger patients. OncoScan microarray analysis, which utilizes both single-nucleotide polymorphism and array-comparative genomic hybridization, is currently the most widely utilized modality for detecting the 11q aberrations, but FISH is a more common test. Although further clinical validation is needed, FCM immunophenotypic characteristics of mnBLL-11q hold promise for clinical diagnosis. Rymkiewicz et al. [16] found that mnBLL-11q usually expresses CD16/CD56/CD38/CD45/CD8/CD43 in FCM and may contribute to the differential diagnosis of BLL, 11q, and BL. In addition, the germinal center marker LMO2 is also a useful marker, since it is typically downregulated in BL and other lymphomas with MYC translocation and expressed in nmBLL-11q [11]. Conversely, although most studies consider 11q23 gain/11q24-qter is mainly absent in other lymphoma entities, its detection should not be considered as a unique tool to diagnose nmBLL-11q cases as some transformed FL may have a similar 11q distortion pattern [17]. In addition to the standard histopathological and immunohistochemical examinations, the FCM, FISH, and other examinations were also performed. Above all, we suggest that this diagnosis should be considered in patients with high-grade B-cell non-Hodgkin lymphomas without MYC rearrangement.

### 3.5 Treatment and prognosis

In terms of treatment and prognosis, although the optimal clinical management remains to be determined, overall, following the Burkitt lymphoma standard treatment regimen, there is a favorable outcome after therapy. The clinical course of nmBLL-11q appears similar to Burkitt lymphoma, but there are few cases reported. In our study, case 1 received R-CTOEP for one cycle and was followed up for 15 months without recurrence and new focus. Although the follow-up time was not long enough, the overall prognosis seems good. Case 2 received R-CHOP for two cycles and then EPOCH for two cycles and was followed up for 7 months without development. These two cases of our study were characterized by case 1 as AIDS patients, presenting with typical Burkitt lymphoma and typical “starry sky” phenomenon, while case 2 as non-AIDS patients presented with the atypical Burkitt lymphoma with small cells and an insignificant “starry sky” phenomenon. In the limited follow-up time, both cases had a good prognosis.
